# Supplementary material for: Are carbon emissions trading and green financial instruments synergistic? -Comprehensive quantitative research based on content analysis
Source: PLoS One. 2024 Mar 7;19(3):e0298601. doi: 10.1371/journal.pone.0298601 (PMC10919601; doi:10.1371/journal.pone.0298601)
Supplement: S1 Data — (ZIP) [file pone.0298601.s001.zip › Data/地级市Z_F工作报告.zip!/╜¡╬≈/╨┬╙α╩╨/└┤╫╘╣1⁄2╓┌║┼í╛┬φ┐╦╩2╛▌═°í┐_2018.docx]

各位代表:

现在,我受市长董晓健同志委托,代表市人民政府向大会报告政府工作,请予审议,并请各位市政协委员和列席会议的同志提出意见。

一、2017年工作回顾

2017年,在省委、省政府和市委的正确领导下,全市上下认真贯彻落实市第八次党代会和市委八届二次、四次全会精神,以务实的精神、落实的办法、坐实的结果,积极应对持续的经济下行压力,全力打好防范化解重大风险、精准脱贫和污染防治攻坚战,奋发有为,开拓进取,全市经济运行质量和效益不断提升,社会发展活力和动力不断增强,人民群众满意度和获得感不断提高。预计(下同)全年实现生产总值1170亿元,增长8.5%;财政总收入144.1亿元,增长5%;固定资产投资1036亿元,增长12.5%;社会消费品零售总额269亿元,增长12%;出口总额92.6亿元,增长10%;实际利用外资4.2亿美元、增长5%,省外2000万元以上项目资金467.5亿元、增长10%;城镇居民人均可支配收入34736元、增长8%,农村居民人均可支配收入16571元、增长9%;居民消费价格指数涨幅控制在3%以内。

(一)聚焦提质增效抓产业,转型步伐越来越快。工业经济持续向好。规模以上工业增加值增长8.3%,主营业务收入增长18%。坚持改造提升传统产业与培育发展新兴产业并重,出台《新余市“中国制造2025”实施方案》《关于加快发展新经济培育新动能的实施意见》和全力支持新钢跨越发展30条措施,深入实施锂电、手机触控、LED、装备制造产业集群三年倍增计划,钢铁、新能源、光电信息、装备制造四大主导产业主营业务收入分别增长15.3%、30.4%、80.2%、19.6%,其中锂电、手机触控和LED等新兴产业分别增长150.6%、40.2%和358.8%。更加注重工业经济的发展质量和效益,规模以上工业实现利润增长57%,其中新钢实现利润增长10倍;工业经济效益综合指数提高59个百分点。充分发挥园区工业主战场作用,新增标准厂房59万平方米,建成产业集群公共服务平台10个,高新区获评省战略性新兴产业集聚区、省锂电新材料产业基地,渝水区新兴工业产业园列为全省产城融合示范区。精准有力帮扶企业,14家企业获产业引导专项资金2.6亿元。市级还贷周转金规模达2.4亿元,帮助企业倒贷300笔、40亿元。赛维重整取得阶段性成果,可望实现突破。新增入规工业企业38户。第三产业日益壮大。实现增加值445亿元、增长9%,新增规模以上服务业企业18户。全市金融机构人民币存、贷款余额分别增长12.3%和4.6%,不良贷款率下降4个百分点,金融风险化解工作成效明显。新余国科在创业板成功上市,泰达长林在“新三板”挂牌,沃格光电主板上市材料获受理,昆仑乐云网络小额贷款公司成功设立。电子商务实现交易额350亿元,增长35%。旅游“双创”工作稳步推进,成功引进复星爱必侬公司运营仙女湖景区,在仙女湖录制的央视七夕晚会反响强烈,乡村旅游蓬勃发展,旅游业接待游客和旅游收入均增长22%。文化产业增加值增长11.4%,占生产总值比重提高到3.75%。第四届国际麻纺博览会、第三届南方农业(工程)机械展销会成功举办。现代农业加快发展。全面落实粮食安全责任,建设高标准农田6.2万亩。52家省级以上龙头企业实现销售收入135亿元,增长4.3%。特色农业势头良好,新余蜜桔产销两旺。农产品加工不断深化,夏布产业列为省示范产业集群。循环农业加快发展,南英2万方特大型沼气工程并网发电,蘑菇产业年处理农业有机废弃物10万吨以上。农村土地经营权流转率达44.3%。创新能力不断提升。全社会研发经费投入15亿元,占生产总值比重达1.4%。新增博士后创新实践基地2家、国家级技能大师工作室1家、省级研发平台5家,建成1家覆盖全市的科技协同创新综合服务平台。加州农业袁隆平院士工作站成立。新增国家高新技术企业13家。争取省级以上科技项目49项、资金1467万元。专利申请量1860件、授权量1018件,每万人发明专利拥有量达2.8件。高新区动力电池产业集群列入科技部第三批创新型产业集群试点。新增百千万人才工程国家级人选1人、省级人选2人,获国务院特殊津贴3人、省政府特殊津贴2人,新增省主要学科学术和技术带头人2人。

(二)强化引擎拉动抓项目,发展后劲越来越足。重点项目成果丰硕。安排重点建设项目199个,总投资增长44%,完成投资198亿元;其中工业项目97个,完成投资112亿元。一批对全市发展具有重大战略意义的项目全面铺开,国省道一级公路绕城改建、城乡供水一体化工程顺利推进;城市供水应急备用水源主体工程基本完工;蒙华公司综合基地落户我市并顺利建成,新余煤储基地项目全面启动;国电投分宜电厂扩建项目全面复工;新余至吉安城际铁路纳入全省城际网规划;分宜通用机场列入省民用机场布局规划。一批对产业转型升级具有重大影响的工业项目加速推进,沃格光电玻璃精加工、赣锋锂业1.5万吨电池级碳酸锂项目建成投产,新钢煤气综合利用高效发电一期工程并网发电,江西雅保5万吨氢氧化锂项目稳步推进。招商引资量质齐升。致力招大引强,突出领导招商、聚焦产业招商、狠抓实体招商,举办或参加各类专题招商推介活动27场,引进悦新大健康产业园、温商五金工具产业基地、科能4亿安时锂电池、牧星现代生态养殖小区、仙女湖航空运营基地等投资10亿元以上项目19个。创新开展资本招商,引进金融创新项目19个、金额18.3亿元。实施“渝郎回乡”工程,共签约项目61个、金额276.4亿元。政策资金争取得力。共向上争取项目266个,增长7.7%。争取上级财政资金59.9亿元、增长10%,其中国家资源枯竭城市转型试点年度绩效考核通过国家评估,获上级补助资金2.86亿元;五河治理工程项目获中央预算内资金1.36亿元。获批财政债券资金75.9亿元,增长66.4%。

(三)围绕舒适宜居抓融合,城乡面貌越来越新。城乡规划更加完善。完成孔目江片区控制性详细规划、海绵城市和综合管廊专项规划等规划编制,创新开展城市设计,实现了农村建房规划建设三级管理网络全覆盖、20户以上村庄规划编制全覆盖。城市功能不断提升。大力实施城市升级三年行动计划,全年安排城建项目27个,完成投资11.7亿元。绿起公园、胜利北路改造、主城区公厕新建等项目顺利建成,11处背街小巷、14个老旧小区、4个城中村基础设施改造全面完成。新增纯电动公交车20辆,新建公交电子站牌100座,实现市、县城区至各乡镇公交全覆盖。第一批20个交通隐患点治理改造全面完成,货车超限超载集中整治深入开展。大数据产业园投入运行,入选中国新型智慧城市创新50强。乡村建设亮点纷呈。乡村两级七项重点工作成效明显,交通信号灯建设实现乡镇全覆盖,“晓康诊所”实现行政村全覆盖,农村生活垃圾治理在全省率先全域通过考核验收,501个新农村建设点完成投资1.5亿元。罗坊生态循环农业小镇、分宜麻纺小镇被评为全省第一批特色小镇,良山镇下保村入选全国环境整治示范村。完成通自然村水泥路建设147.4公里、公路安全生命防护工程254公里。在全省率先完成新一轮农村电网升级改造“两年攻坚战”工程。

(四)突出关键环节抓改革,体制机制越来越活。35项重点改革项目年度任务全面完成。行政管理体制改革取得新成效。行政审批标准化体系全面建立,政务服务更加高效便捷。“双随机一公开”监管工作改革全面推进,市直行政执法单位全部接入全省统一监管平台。机构编制管理改革扎实推进。在全省率先开展的城市执法体制改革试点工作成效明显,各项改革任务全面完成。经济体制改革实现新突破。供给侧结构性改革深入推进。大力实施降成本优环境专项行动,输配电价改革为企业降低用电成本每度4分以上,规模以上工业每百元主营业务收入成本降低2.2元,全年为企业减负30.2亿元。房地产去库存工作成效显著,中心城区新建商品房住宅去化周期缩短至7.6个月。新宜吉六县跨行政区转型合作试验区建设稳步推进。积极化解政府性债务,超额完成年度目标。市本级行政事业单位闲置资产清理整合盘活工作有序开展,市属国企公司制改革全面完成,三大农垦场均组建了国有资本投资营运公司。社会管理体制改革迈出新步伐。社会组织和企业信用体系建设基本完成,公职人员信用评级结果运用力度加大。社区数字化综合服务管理平台升级完善,新增4个社区上线运行。出租汽车行业改革稳步推进。

(五)立足和谐共生抓环保,生态环境越来越美。环境治理力度加大。大力实施“保家行动”,集中开展畜禽养殖、水库退养、工业污染水环境专项整治,关停拆迁禁养区养殖场6339家,333座水库、2293座山塘全部退出承包养殖,废水排放不达标的53家企业全部整改。11个国控、省控监测断面有7个达到Ⅲ类水质标准,消灭劣V类水工作初见成效。大力开展“蓝天行动”,集中实施“四尘”“三烟”“三气”综合整治,城区空气质量优良率达86.2%。土壤污染防治工作方案编制完成,原前卫化工厂场地土壤综合治理与修复工作积极推进。生态保护成效明显。推行分布式为主、集中式为辅的城乡污水处理新模式,89座村镇生活污水处理设施建成投运。加大工业企业污染防治力度,新钢焦炉除尘(二期)、6号高炉和4号烧结机除尘改造设施建成投运。完成仙女湖和孔目江水源地规范化建设及防护工程,饮用水源地周边环境风险源全部消除。强化环境执法监管,先后开展环保专项行动22项,依法关停取缔“散乱污”企业57家。新增造林4.7万亩。制度体系更加完善。生态保护红线、城镇开发边界划定工作扎实开展,永久基本农田划定工作全面完成。河长制工作体制机制不断健全,孔目江流域生态补偿成效明显,全国水生态文明城市建设试点通过验收。第一部实体地方性法规《仙女湖水体保护条例》,已提交市人大常委会审议。领导干部自然资源资产审计工作走在全省前列。分宜县被确定为全省绿色低碳示范县,渝水区列为全省生态文明示范区。

(六)坚持不忘初心抓民生,惠民成果越来越实。45件民生实事基本完成。脱贫攻坚更加扎实。贫困人口识别退出进一步精准,全市共有建档立卡贫困人口8634户21003人。出台《关于进一步加强社会扶贫工作的实施意见》,共安排419个单位、所有在职领导干部、“两代表一委员”、26个商(协)会、1200家民营企业与贫困对象结对帮扶,实现贫困村、贫困户帮扶全覆盖。创新开展“播爱使者”活动,帮助单身贫困群众解决婚恋问题。深入推进八大扶贫工程,配套财政扶贫资金3807.3万元,3479名贫困人口实现脱贫。就业创业更加充分。新增城镇就业2.2万人、就业率达96.6%,新转移农村劳动力1.6万人。新发放创业担保贷款4.2亿元,直接扶持2411人创业,带动10544人就业。社会保障更加有力。未参保城镇大集体企业退休职工养老生活月补助标准由375元提高到395元,全民参保登记计划全面启动。被征地农民参加基本养老保险工作强力推进,2018年可按规定发放待遇。城镇居民医保和新农合正式并轨运行,跨省异地就医结算系统全面启用。为11万名65岁以上老人购买了意外伤害保险,为2.6万名80岁以上老人发放了高龄补贴。建成颐养之家603个,入家老人7597人,实现全市行政村颐养之家全覆盖;市养老福利中心建成运营。发放廉租房租赁补贴5268户、856.6万元,改造棚户区5488户、垦区危旧房4800户。公共事业更加繁荣。在全省率先通过义务教育基本均衡发展的国家认定,乡村小规模学校适度集中办学试点成效明显,成功争取清华大学和北京大学在我市设立自主招生考点。城市公立医院综合改革获国务院通报表扬,被确定为首批国家级示范城市。成功入选国家文化消费试点城市,获评全国未成年人思想道德建设工作先进城市,国家食品安全示范城市创建、国家公共文化服务体系示范区后续建设工作稳步推进。成功举办环鄱阳湖仙女湖站国际自行车大赛、环仙女湖国际马拉松等赛事,第十三届全运会上获金牌2枚。社会治理更加有序。“共产党员示范市”创建工作全面启动。“新余有爱”志愿服务品牌深入人心。商(协)会建设成效显著。积极推行领导包案化解社会矛盾工作机制,群众诉求渠道更加畅通。法治新余和平安新余建设深入推进,国家安全人民防线进一步加强,没有发生较大及以上安全事故,没有发生重大群体性事件,没有发生重特大刑事案件,全市社会和谐稳定。统计、海关、检验检疫、民族宗教、外事侨务、涉台事务、档案、地方志、公积金、机关事务、防灾减灾、妇女儿童、青少年、老龄、计生、科普、气象、人防、邮政通讯、烟草、盐业、海事、民兵预备役、社科研究、红十字会人道援助和慈善、残疾人事业等工作取得新成绩。

各位代表!过去的一年,我们扎实推进“两学一做”学习教育常态化制度化,认真学习宣传贯彻党的十九大精神,“四个意识”更加牢固;我们全面推进依法行政,严格落实法治政府建设工作要点,行政决策科学化、民主化、法治化水平进一步提升;我们不断加大对“为官不为”等问题的治理力度,严肃问责了一批懒政怠政干部,机关作风持续好转,行政效能不断提高;我们深入开展“红包”“违插”等问题专项治理,突出重点领域、重点岗位和关键环节的风险防控,严肃查办了一批违法违规案件,政府自身建设不断加强。

过去的一年,克服了很多困难,化解了不少矛盾,成绩的取得实属不易。这得益于中央、省委省政府和市委的坚强领导,得益于市人大的法律监督和市政协的民主监督,得益于社会各界和全市广大人民群众的积极作为。在此,市人民政府向全市广大工人、农民、知识分子、干部和历任老领导、老同志,向各民主党派、工商联、无党派和社会各界人士,向驻市人民解放军、武警官兵和公安干警,向驻市中央和省属单位,致以崇高的敬意!向所有关心、支持新余发展的同志们、朋友们,表示衷心的感谢!

回顾过去一年的工作,我们清醒地看到,与人民群众日益增长的美好生活需要相比,全市经济社会发展不平衡不充分的问题依然突出:经济发展总量偏小,发展质量和效益还不高,创新意识和能力还不强,新兴产业支撑作用还有待提升;政府性债务化解难度不小,财政收支平衡压力较大;生态环境保护任重道远,呵护青山绿水、蓝天白云还有很多工作要做;城乡基础设施还有不少短板,公共服务供给水平与群众需求仍有不少差距;少数干部担当勇气、创新精神和实干劲头还不足,干部作风建设还需持续加强,等等。对此,我们一定认真对待,切实加以解决。

二、2018年工作安排

2018年是贯彻党的十九大精神的开局之年,是改革开放40周年,是决胜全面建成小康社会、实施“十三五”规划承上启下的关键一年。政府工作的总体要求是:全面贯彻落实党的十九大、中央经济工作会议和省委十四届五次全会精神,以习近平新时代中国特色社会主义思想为指导,按照高质量发展的要求,坚持稳中求进工作总基调,坚持新发展理念,坚持以供给侧结构性改革为主线,突出抓重点、补短板、强弱项,统筹推进稳增长、促改革、调结构、惠民生、防风险各项工作,促进经济社会持续健康发展。

全市经济社会发展的主要预期目标是:生产总值增长8.5%;财政总收入增长7%;规模以上工业增加值增长8.6%,主营业务收入增长8%;固定资产投资增长11%;社会消费品零售总额增长11.5%;外贸出口总额增长3%;实际利用外资增长6%,省外2000万元以上项目资金增长7%;城镇居民和农村居民人均可支配收入分别增长8%和8.5%;居民消费价格指数控制在3%左右。节能减排完成省下达的计划任务。

围绕上述目标,重点做好以下工作:

(一)出好“三记硬招”,在工业经济上奋力崛起。坚持工业立市、工业强市不动摇,把创新作为引领发展的第一动力,把实体经济的重点放在制造业上,加快打造全省产业转型升级典范。

出好新旧动能转换招。积极申报“中国制造2025”国家级示范区,加快培育一批具有创新能力的排头兵企业,着力构筑新型制造业体系。全面落实支持新钢跨越发展30条措施,推动新钢实施5-10个产业延伸合作项目,加快发展非钢产业。积极稳妥做好赛维重整工作。全面完成产业集群三年倍增计划,力争锂电、手机触控产业全年主营业务收入均达220亿元,LED和装备制造产业均达200亿元。充分发挥高新区军民融合智能装备制造产业基地的示范带动作用,加快推进军民融合产业深度发展。

出好实体经济帮扶招。深入开展降成本优环境专项行动和企业精准帮扶,全面落实各项惠企政策,切实提高企业全要素生产率。用活用好产业引导专项资金和工业发展基金,支持重点企业发展和产业集聚壮大。出台金融机构支持地方经济发展考核奖励办法,充分调动金融机构信贷投放积极性。不断完善园区基础设施,加快建设配套服务体系,积极盘活园区闲置资产,切实提高标准厂房利用率。确保新增入规工业企业38户以上。

出好创新驱动引领招。积极争创国家创新型试点城市,深入实施创新驱动“3315”工程,力争全社会研发经费投入占生产总值比重达1.5%。新增国家高新技术企业10家,申报省级以上项目40项,实施市级科技重点项目20项,新增市级科技研发平台6家。力争专利申请量达1900件,每万人发明专利拥有量达2.9件。推动企业省级以上科研平台向全市开放,组建锂电、钢铁产业技术创新联盟。加大人才引进力度,着力引进战略性新兴产业紧缺人才和高层次人才。

(二)瞄准“三个提升”,在三产繁荣上精准发力。围绕提升供给质量和水平,加快推动服务业发展壮大,确保新增入规服务业企业10户以上,实现增加值增长9%。

推动生产性服务业向专业化提升。引导金融机构创新信贷产品,优化投放结构,加大对“三农”和小微企业支持力度。全面完成产业集群公共服务平台建设三年行动计划,不断提升平台服务水平和运营效益。加快发展现代物流业,基本建成陆路口岸查验区和电子口岸。大力发展工业设计,引领产业向价值链高端提升。充分发挥国际麻纺博览会等会展平台作用,不断提升产业影响力和竞争力。

推动生活性服务业向品质化提升。深入推进国家全域旅游示范区创建,着力抓好市、县、乡三级节点基础设施建设。全力推进仙女湖创建国家5A级景区,加快提升景区品质和形象。大力推动新余国际广场、上亿广场等重点商贸项目建成投运,加快建设跨区域农产品流通基础设施。大力培育文化龙头企业,加快发展文化产业新兴业态,力争产业增加值占生产总值比重达3.9%。

推动新兴服务业向规模化提升。加快发展农村电商,建成10个乡镇电商服务中心和50个村级电商服务站。充分发挥大数据产业园带动效应,抓好云计算中心二期建设,吸引更多IT企业落户,大力培育智慧经济。培育壮大家政服务龙头企业,加快推进家政服务业市场化、产业化步伐。积极争取社区健康养老管理体系建设省级试点,努力打造健康养老服务品牌。积极发展体育产业,加快推进体育与旅游、文化等产业融合发展。

(三)做优“三篇文章”,在投资增效上敢抢敢拼。大力实施项目带动战略,全力做好建项目、引项目、融资金“三篇文章”,在项目扩量和提质上下工夫、出实招、求实效。

做优重点项目建设文章。全年计划安排重点项目200个、年度计划投资200亿元,其中工业项目投资额占比达到50%以上。不断完善市领导挂点、市直单位帮扶机制,继续开展分季度、分县区重大项目集中开工活动,加快推进重点项目管理数字化、信息化。重点抓好投资过10亿元的“三个一批”重大项目建设,全力推进国省道一级公路绕城改建、蒙华铁路新余段、国电投分宜电厂扩建等一批基础设施项目建设,加快推进大唐新余二期扩建项目,做好新余至吉安城际铁路和分宜通用机场项目前期工作,建成城市供水应急备用水源工程;切实抓好东鹏化工2.5万吨锂盐、江西雅保5万吨氢氧化锂、科能4亿安时锂电池等一批工业项目建设;加快推进悦新大健康产业园、南英垦殖场综合开发、分宜麻纺小镇、河下渔仙小镇等一批现代服务业项目建设。

做优招商引资提质文章。立足四大支柱产业和特色优势产业,围绕大企业紧盯大项目,实行产业链精准招商、配套招商,组织参加23场招商推介活动,推动招大引强实现再突破。鼓励园区龙头企业围绕产业链、产业群自主招商,吸引投资商集聚发展。深入推进赣企入余、“渝郎回乡”,发挥商会招商、乡贤招商更大作用,以感情待人,以事业留人。出台全市统一的招商引资优惠政策,抓好政策兑现落实,打造透明、可预期的投资环境。

做优融资渠道拓宽文章。增强资本市场服务实体经济功能,积极有序发展股权融资,鼓励优质企业发行债务融资工具。充分运用人保财险“政融保”融资模式,着力破解新型农业经营主体融资难、融资贵问题。引导企业加快直接融资步伐,出台企业上市三年行动计划,推动沃格光电在主板上市。准确研判政策走向,加强项目谋划储备,力争争取上级资金增长10%以上。

(四)走好“三步路子”,在融合发展上协调推进。坚持高标准规划、高质量建设、高效能管理,全力推进城乡基础设施建设,不断提升城乡发展质量,让城市更有品味、农村更有活力。

走好城市品质提升路。完成城市近期建设规划和南部片区空间规划,加快高铁新区控规修编工作,全面落实海绵城市、地下综合管廊等各类专项规划,深入推进城市设计,谋划好城市“美”的空间。加大存量土地盘活力度,努力解决土地供给不足与城市加快发展的矛盾。加快推进城市升级三年行动计划,全年安排市本级城建项目32个、年度计划投资12.5亿元,其中城区生活污水治理、袁河公园等新建项目23个,中洲城区棚户区改造、孔目江湿地公园三期等续建项目9个。深入实施交通安全隐患治理工程,大力推进5条背街小巷、2个老旧小区基础设施改造,切实改善城区居民生活环境。

走好美丽乡村振兴路。按照产业兴旺、生态宜居、乡风文明、治理有效、生活富裕的总要求,持续开展乡村两级七项重点工作。扎实推进城乡公厕新建改造,全力推进农村环境综合整治,建设好整洁美丽的“后花园”。坚持政府引导、企业主体、社会参与的运作模式,加快编制特色小镇建设专项规划,加大项目谋划和推介力度,引进更多社会资本建设好全市第一批8个特色小镇,力争成功申报国家级、省级特色小镇各1个。大力推进“四好农村路”建设,不断完善城乡路网体系,完成县道升级改造34.2公里、安防工程建设180公里。深入推进城乡公交一体化,逐步将农村客运纳入公共交通服务体系。全面建成城乡供水一体化工程,实现50户以上自然村供水管网全覆盖。扎实推进分宜小康用电示范县建设。

走好现代农业发展路。坚持质量兴农、绿色兴农,加快推动农业供给侧结构性改革,促进农村一二三产业融合发展。稳步推进农村集体产权制度改革,抓好土地整治补充耕地工作,引导土地集中连片流转,发展多种形式适度规模经营。引进培育龙头企业,做大做强特色农业,加快发展休闲农业,着力打造一批生态艺术性田园游居综合体。深入开展绿色生态农业“十大行动”,大力推广罗坊区域生态循环农业模式,加快建设一批绿色农产品规模化生产基地。加大农产品宣传推广力度,着力打造一批有影响力、有竞争力的品牌。认真开展第三次全国土地调查。

(五)下活“三枚棋子”,在改革创新上全力攻坚。坚持向改革要动力,以改革促发展,继续在重要领域和关键环节下好改革“先手棋”,全力打造优质高效的发展环境。

下活行政管理体制改革棋。继续推进“一窗式”审批服务改革,基本实现“前台综合受理、后台分类审批、统一窗口出件”。加快推进“互联网+政务服务”,积极开展“一次不跑”政务服务事项改革。全面深化商事制度改革,重点抓好“证照分离”先行先试工作,稳妥推进电子营业执照和企业登记全程电子化。巩固城市执法体制改革成果,全面理顺工作职责,加快形成权责明晰、高效有序的城市管理模式。坚持问题导向,深入推进开发区改革和创新发展。

下活经济体制改革棋。积极推动新宜吉六县跨行政区转型合作试验区建设,着力推进试验区工作协商机制的建立,加快实施一批实质性合作项目。认真落实赣西经济转型发展战略,积极谋划一批我市牵头的合作事项,全力推动三市合作项目落地见效。加快化解政府性债务,全面完成年度任务。推进市以下财政事权与支出责任划分改革,加快理顺市与县(区)共同财政事权。完善国有资产集中统一监管体制,推动国有企业做强做优做大,做好国有企业社会职能承接工作。

下活社会管理体制改革棋。稳步推进价格改革,进一步减少政府定价项目。深入推进社会信用体系建设,完善社会组织与企业信用体系,强化信用评级结果运用,探索开展自然人信用数据征集工作。切实加强社区治理体系建设,努力推动社会治理重心向基层下移。稳妥推进殡葬改革。

(六)做到“三个坚持”,在绿色发展上持之以恒。树立和践行绿水青山就是金山银山的理念,像对待生命一样对待生态环境,坚决打好污染防治攻坚战,还自然以宁静、和谐、美丽。

坚持以过硬的举措开展环境整治。推进“保家行动”常态化,全面完成可(限)养区养殖场生态化改造和山塘、水库水质达标整治工作,加快畜禽养殖废弃物资源化利用步伐。抓好仙女湖、袁河、孔目江、袁惠渠等流域的污染源治理、生态修复和饮用水源地综合整治工作,建成村镇生活污水处理设施61座,坚决消灭劣V类水体,确保集中式生活饮用水源地水质达标率为100%。深入开展“蓝天行动”,持续加大工业大气污染整治力度,加强“四尘”“三烟”“三气”联合防治,确保空气质量优良率、PM2.5等约束性指标控制在省政府下达的目标范围内。完善土壤常规监测体系,有效防范土壤污染。

坚持以最严的标准加强生态保护。坚决把好项目准入关,从源头上防止环境污染和生态破坏。持续开展环保专项行动,进一步强化重点行业和重点区域的环境执法监管,提高环境突发事件的应对能力,确保环境安全。严格实施生态红线管制,打好环境执法组合拳,严肃查处环境违法行为,促进环境守法成为常态。

坚持以完善的制度构建生态屏障。严格落实环境保护党政同责、一岗双责责任制,加快编制自然资源资产负债表,强化生态文明建设工作考核。积极推进环境污染第三方治理,拓宽合同环境服务、碳排放交易试点领域。建立健全跨区域联合治污工作机制,推动水、大气污染防治实现跨流域、跨县(区)联防联控。积极推广绿色建筑和装配式建筑,加快推进生活垃圾分类管理和餐厨垃圾资源化利用,深入开展创建节约型机关、绿色家庭、绿色学校、绿色社区和绿色出行等行动。

(七)织密“三张网络”,在民生改善上久久为功。坚持在发展中保障和改善民生,多谋民生之利、多解民生之忧,全力办好40件民生实事,让改革发展成果更多更公平惠及全市人民。

织密民生底线保障网。坚决打好精准脱贫攻坚战,充分发挥社会力量作用,切实抓好八大扶贫工程,力争有条件的行政村建设50千瓦光伏电站,确保34个省级贫困村光伏扶贫扩面工程顺利并网,保证现行标准下的脱贫质量既不降低标准、也不吊高胃口。坚持更加积极的就业创业政策,大力推进创新创业平台建设,努力实现更高质量和更充分的就业。加强社会保险扩面征缴,继续实施全民参保登记计划。完善各项救助保障制度,实现城乡低保应保尽保。巩固完善农村“颐养之家”建设,加快推进城区居家和社区养老平台建设,不断满足城乡居民的养老服务需求。

织密社会事业繁荣网。坚持教育优先发展战略,加快推进高新一中、北湖小学、松山小学等学校建设,着力解决“择校热”“大班额”等突出问题;努力提升学前教育普惠水平,探索推行小升初招生改革,积极应对高考制度改革,加快发展社区教育。深入推进健康新余建设,继续巩固公立医院综合改革成果,加快推进市中医院迁建、市妇幼保健院改扩建等项目建设,着力解决“看病难、看病贵”问题。努力创建国家卫生城市。全力推进国家文化消费试点城市创建,继续做好国家公共文化服务体系示范区后续建设工作。广泛开展全民健身活动,力争在第十五届省运会取得优异成绩。关心支持妇儿、老龄事业,发展残疾人事业和慈善事业。加强国防后备力量和人民防空建设,推进“双拥”和优抚安置,巩固和发展军政军民团结。

织密社会治理和谐网。坚决打好防范化解重大风险攻坚战,做好重点领域风险防范和处置,严厉打击违法违规金融活动,加强薄弱环节监管制度建设,促进形成金融和实体经济、金融和房地产、金融体系内部的良性循环。启动第六届全国文明城市创建工作。加强社会主义法治文化和公共法律服务平台建设,加快构建城乡立体化、信息化社会治安防控体系,依法打击和惩治黄赌毒黑拐骗等违法犯罪活动,保护人民人身权、财产权、人格权。大力实施食品安全战略,加快推进国家食品安全示范城市创建工作,让人民吃得放心。牢固树立安全发展理念,严格落实安全生产责任制,坚决遏制重特大事故发生,提升防灾减灾救灾能力。畅通信访渠道,加强预防和化解社会矛盾机制建设,提高对各类突发事件的预警预防和应急处置能力。

各位代表!中国特色社会主义进入新时代,对政府工作提出了新的更高要求。我们将牢固树立“四个意识”,夙夜在公,埋头苦干,在新时代担当新使命,以新作为创造新业绩。

守住法治底线,始终做到依法行政不越界。认真落实“三重一大”报告和政府法律顾问制度,抓好重大行政决策的合法性审查,确保政府行为于法有据。加大执法活动监管力度,严格落实行政执法评议考核制度,进一步规范执法行为。持续推进政务公开,主动接受人大法律监督、政协民主监督和社会各界监督,认真办理人大议案、政协提案及其意见建议。

牢记使命担当,始终做到奋发有为不懈怠。扎实开展“不忘初心、牢记使命”主题教育,坚决反对形式主义、官僚主义,持续纠“四风”改作风。大力开展调查研究,不断加强学习和实践,培养专业能力,弘扬专业精神。坚持把雷厉风行和久久为功有机结合起来,勇于攻坚克难,以钉钉子精神做实做细做好各项工作。着力治理庸懒散,坚决向不作为、慢作为、乱作为等问题亮剑,确保政令畅通、令行禁止,不断优化发展环境。严格落实正向激励、澄清保护和容错纠错机制,给干事者鼓劲,为担当者撑腰。

永葆廉洁本色,始终做到秉公用权不任性。坚持挺纪在前,全面履行党风廉政主体责任和一岗双责,认真落实八项规定。抓住重点领域和关键环节,加强对项目招投标、政府性采购、土地出让等工作的监督管理,严肃查处侵害群众利益的不正之风和腐败问题。坚持用制度管权管事管人,严防权力失控、决策失误、行为失范,持续推动干部清正、政府清廉、政治清明。

各位代表!站在新时代,我们豪情满怀;展望新征程,我们信心倍增。让我们紧密团结在以习近平同志为核心的党中央周围,在省委、省政府和市委的坚强领导下,奋力攻坚、奋勇向前,在建设“三区两典范”、再铸“工小美”辉煌的道路上迈出更加坚实的步伐!
